# Viral Findings in Adult Hematological Patients with Neutropenia

**DOI:** 10.1371/journal.pone.0036543

**Published:** 2012-05-03

**Authors:** Lars Öhrmalm, Michelle Wong, Carl Aust, Per Ljungman, Oscar Norbeck, Kristina Broliden, Thomas Tolfvenstam

**Affiliations:** 1 Department of Medicine, Solna, Infectious Disease Unit, Center for Molecular Medicine, Karolinska Institutet, Karolinska University Hospital, Stockholm, Sweden; 2 Division of Hematology, Department of Medicine, Karolinska Institutet, Stockholm, Sweden; 3 Hematology Centre, Karolinska University Hospital, Stockholm, Sweden; University Hospital San Giovanni Battista di Torino, Italy

## Abstract

**Background:**

Until recently, viral infections in patients with hematological malignancies were concerns primarily in allogeneic hematopoietic stem cell transplant (HSCT) recipients. During the last years, changed treatment regimens for non-transplanted patients with hematological malignancies have had potential to increase the incidence of viral infections in this group. In this study, we have prospectively investigated the prevalence of a broad range of respiratory viruses in nasopharyngeal aspirate (NPA) as well as viruses that commonly reactivate after allogeneic HSCT.

**Methodology/Principal Findings:**

Patients with hematological malignancies and therapy induced neutropenia (n = 159) were screened regarding a broad range of common respiratory viruses in the nasopharynx and for viruses commonly detected in severely immunosuppressed patients in peripheral blood. Quantitative PCR was used for detection of viruses. A viral pathogen was detected in 35% of the patients. The detection rate was rather similar in blood (22%) and NPA (18%) with polyoma BK virus and rhinovirus as dominating pathogens in blood and NPA, respectively. Patients with chronic lymphocytic leukemia (CLL) (p<0.01) and patients with fever (p<0.001) were overrepresented in the virus-positive group. Furthermore, viral findings in NPA were associated with upper respiratory symptoms (URTS) (p<0.0001).

**Conclusions/Significance:**

Both respiratory viral infections and low titers of viruses in blood from patients with neutropenia were common. Patients with CLL and patients with fever were independently associated to these infections, and viral findings in NPA were associated to URTS indicating active infection. These findings motivate further studies on viruses' impact on this patient category and their potential role as causative agents of fever during neutropenia.

## Introduction

Reactivation of viruses in blood as well as viral respiratory tract infections has a significant impact on the morbidity and mortality of allogeneic hematopoietic stem cell transplant (HSCT) recipients [1]–[4]. Although a rather high prevalence of respiratory viruses in neutropenic patients with fever is reported [4], [5], the impact of viral infections in the non-transplant setting of neutropenic hematological patients is less studied. In addition, reactivation of cytomegalovirus (CMV) is reported in hematological neutropenic patients that have not undergone allogeneic HSCT [6]–[8]. Fever is often the only sign of infection in the immunocompromised patient, and since establishing the cause of fever in neutropenic patients is difficult, empiric administration of broad spectrum antibiotics is in common usage [9], [10]. This strategy has substantially decreased the mortality rate in this patient group, but bacterial infection is documented in only approximately one-third or fewer of the febrile episodes [11]–[14]. Most patients with hematological malignancies remain at home between courses of chemotherapy, including neutropenic phases, and are thus exposed to all common pathogens circulating in the society. Consequently, the epidemiology of these infections in hospitalized patients mirrors that in outpatients [15].

Here, in a setting of neutropenic hematological patients, other than allogeneic HSCT recipients, we prospectively evaluated the presence of a broad range of respiratory viruses in nasopharyngeal aspirate (NPA) as well as viruses that commonly reactivate after allogeneic HSCT.

## Materials and Methods

### Ethics Statement

Patients were, after giving their informed written consent, eligible for enrollment. The study was approved by The Regional Ethical Review Board in Stockholm (www.epn.se).

### Study population

During a 26-month period (Jan 2008–Feb 2010) adult patients with hematological disorders who presented at the Karolinska University Hospital, Stockholm, with neutropenia were asked to participate in this study. Patients having undergone allogeneic hematopoietic stem cell transplantation (HSCT) within the previous two years and patients admitted to the Intensive Care Unit were excluded from the study.

### Sampling procedure

Patients with neutropenic fever were sampled for routine microbial cultures upon admission or at the onset of fever during a hospitalization. The additional study samples for detection of viruses in blood and nasopharyngeal aspirates (NPA) were collected within 72 hours of fever onset. Upon routine medical appointments, unselected afebrile neutropenic patients were prospectively sampled for the study samples only. Within 6 hours from sampling, each NPA was stored at −80°C before analysis by quantitative real-time PCR (qPCR). Serum was used for all blood assays except those for cytomegalovirus (CMV) and adenovirus (AdV), where whole blood and plasma were used, respectively.

### Extraction and qPCR methods

Blood specimens for detection of Epstein-Barr virus (EBV), CMV, and AdV were extracted and analyzed by qPCR as described [16]. For the other qPCRs, total viral nucleic acids were extracted with QIAamp MinElute Virus Spin Kit (QIAGEN, US) according to the manufacturer's protocol with 200 µL of specimens eluted into 50 µL of extract. The analysis of BK virus (BKV) is described elsewhere [Multiplex real-time PCR assay for simultaneous detection of cytomegalovirus, Epstein-Barr virus, adenovirus, and BK polyomavirus, Öhrmalm et al. In manuscript]. The qPCR for parvovirus B19 (B19) was performed on an ABI 7500 Real-Time PCR System (Applied Biosystems) and carried out in a total 50-µL reaction mixture including 25 µL of TaqMan Universal PCR Master Mix (Applied Biosystems) and 5 µL of template (Table 1). The NPAs were analyzed on the same ABI System described above. However, for RNA viruses, a MultiScribe and RNase Inhibitor Mix (Applied Biosystems) was used. Newly developed primers and probes are described in Table 1, whereas for human rhinovirus (HRV) [17], human enterovirus (HEV) [18], influenza A virus (IFA) [19], influenza B virus (IFB) [20], respiratory syncytial virus (RSV) [21], and parainfluenza virus type 1 (PIV1) [22], the design is described elsewhere. Quantification of viruses in blood was achieved by comparing with plasmids of known concentrations. Virus detection in nasopharynx was however judged qualitatively as NPA was used as starting material. For CMV, EBV, and AdV the dynamic range was 500 to 2×10^8^ copies/mL. Values of detectable viral copies below this range were given as “<500". For BKV and B19, the lower part of the dynamic range was 5 copies/reaction. Values of detectable viral copies below 250 copies/mL blood were considered as “<250.

**Table 1:** Description of newly developed primers and probes used in the qPCR assays.

Virus	Primer/Probe	Sequence (5′-3′)	Conc. (nM)	PCR program
hCoV	FW	YGATAAAGCTGTAGCTCGCAAACT	900	48°C for 30 min→95°C for 10 min→40 cycles of 15 sec at 95°C and 60 sec at 60°C
		ACGCGACCGTGCTGTAGC		
		GATCATGAGATATCTGTTCAGAAGAAAATT		
		CAGGGAATCATCTGTTCAAAAGAA		
	REV	GATTATCCAATTTACGAACCATGCTA	900	
		GCTTGATTATCTAACTTACGCACCATACTA		
		ACATATCCAAACGTCTTAACATTCCA		
		AAACGTCGGAGCATGCCA		
	PRO	VIC-GATAAGAAGAGTAARGTTGTTTC-MGB	225	
		VIC-TGGCTGAACAAGCTG-MGB		
hMPV	FW	AAAGCATTAGGCTCATCCTCTACAG	300	
		AAAGCTTTAGGCTCATCTTCAACAG		
		AAAGCATTAGGCTCATCATCTACAGG		
	REV	ATTGTTAGATGACCTGGCAATGAC	300	
		GTTGGATGACCTGGCAATGAC		
		TGTTGGATGATCTGGCAATGAC		
		TGTTGTTAGATGATCTGGCAATGAC		
		TGTTAGATGACCTGGCGATGAC		
	PRO	NED-AGCAAAGCAGAAAGT-MGB	225	
IFB	PRO^a^	6FAM-CAGATCTGTGCAGTTGAG-MGB		
PIV2	FW	TTACCTAAGTGATGGAATCAATCGC	300	
	REV	TCTTTYTCAGAYCTTGTAGCTACATAGCA	300	
	PRO	NED-AAGCTGTTCAGTCACTGC-MGB	175	
PIV3	FW	TCCCCATGGACATTCATYGTT	900	
	REV	TGGCAYAGCAARTTACAATTAGGAA	900	
	PRO	NED-TGCCATGTCCATTTTA-MGB	175	
PIV4	FW	CCAGTCAAATCAAYTGCCTCYG	900	
	REV	GATCTCTRATGCATAGTTCGCAAATT	900	
	PRO	NED-CATGTGGAAGATGTCC-MGB	225	
B19	FW	CCAGGCGCCTGGAACA	50	50°C for 2 min→95°C for 10 min→40 cycles of 15 sec at 95°C and 60 sec at 60°C
	REV	TCGGAGGAAACTGGGCTTC	300	
		TTCGGAGGAAACTGAGCTTCC		
	PRO	6FAM-CGGGACCAGTTCAGGA-MGB	250	

**NOTE.** Conc, concentration; FW, forward; REV, reverse; PRO, probe; hCoV, human coronavirus; hMPV, human metapneumovirus; IFB, influenza B virus; PIV, parainfluenza virus; B19, parvovirus B19.

a Probe modified, otherwise as described by Tiveljung et al [20].

### Flow cytometry

In 99 patients where peripheral blood mononuclear cells were isolated phenotype characterization was made by staining with two sets of antibodies, BD Multitest (BD Biosciences, Sweden), against the following cell surface markers; Set 1: CD3-FITC, CD8-PE, CD45-PerCP, CD4-APC; Set 2: CD3-FITC, CD16/CD56-PE, CD45-PerCP, CD19-APC according to the manufacturer's instructions. TruCount tubes (BD Biosciences) were used for an absolute cell count.

### Definitions and diagnostic criteria

Neutropenia was defined as an absolute neutrophil count ≤500/mm^3^. Fever was defined as a single temperature reading ≥38.5°C or >38.0°C that persisted for 1 hour. Upper respiratory tract symptoms (URTS) were defined as onset of rhinorrhea, sneezing, nasal congestion, cough, or hoarseness. For a diagnosis of coagulase-negative staphylococci bacteremia, at least two positive blood cultures were required.

### Data collection and statistical analysis

Clinical and routinely collected laboratory data were extracted from all patients' medical records. Univariate analyses were performed using Fischer's exact test for categorical data. Non-categorical variables were compared with the Mann-Whitney U-test. Forward conditional binary logistic regression analysis was performed with presence of virus as the dependent variable and all parameters described in Table 2 with a p-value <0.10 as covariates. All tests were two-sided with p-value of <0.05 considered significant. Software products used were Prism 5.00 for Windows and PASW Statistics 18.

**Table 2:** Characteristics and laboratory data on neutropenic patients with and without viral findings.

	Any virus		No virus		
Characteristics	(n = 56)		(n = 103)		OR (95%CI)^a^
No. of females (%)	21	(38)	44	(43)	0.80 (0.41–1.6)
Age, [years], median (range)	62	(20–86)	60	(25–86)	n.s.
Underlying disease (%)					
Acute leukemia/MDS	19	(34)	51	(50)	0.52 (0.27–1.0)†
Chronic myeloid leukemia	0	(0)	1	(1)	0.60 (0.02–15)
Chronic lymphocytic leukemia	6	(11)	1	(1)	12 (1.4–104)**
Non-Hodgkin lymphoma	25	(45)	29	(28)	2.1 (1.0–4.1)†
Myeloma	4	(7)	16	(16)	0.4 (0.13–1.3)
Hodgkin's disease	1	(2)	3	(3)	0.61 (0.06–6.0)
Others	1	(2)	2	(2)	0.92 (0.08–10.4)
No. of autologous HSCT (%)	2	(4)	27	(26)	0.10 (0.02–0.46)***
Latest immunosuppressive treatment (%)					
Monoclonal antibodies^b^	15	(27)	10	(10)	3.4 (1.4–8.2)**
Antineoplastic chemotherapy	49	(88)	98	(95)	0.36 (0.11–1.2)
Steroids	22	(39)	19	(18)	2.9 (1.4–5.9)**
Days from treatment to sampling	12	(1–1064)	13	(2–632)	n.s.
Cell count^c^ [/mm^3^], median (range)					
Leukocytes	200	(<100–1870)	400	(<100–3000)	n.s.
Neutrophils	<100	(<100–500)	<100	(<100–500)	n.s.
Lymphocytes	127	(14–18552)	202	(2.3–1332)	n.s.
CD4^+^ T cells	38	(0.06–769)	93	(1.4–606)	*
CD8^+^ T cells	45	(1.1–1568)	51	(0.14–537)	n.s.
B cells	0.2	(0.02–15061)	0.4	(0.02–439)	n.s
NK cells	11	(1.0–96)	16	(0.32–196)	n.s.
Monocytes	21	(0.03–495)	86	(0.03–1337)	n.s.
Prophylactic aciclovir (%)	21	(38)	58	(56)	0.47 (0.24–0.91)*
Presence of fever	52	(93)	71	(69)	5.9 (2.0–18)***
Bacterial infection^d^	15	(27)	26	(25)	1.1 (0.52–2.3)

**NOTE.** Characteristics are presented in number and percentage of each group. Medians are followed by range. OR, odds ratio; CI, confidence interval; MDS, myelodysplastic syndrome; NPA, nasopharyngeal aspirate.

a† , p≤0.10;

* , p<0.05;

** , p<0.01;

*** , p<0.001; ns, not significant.

b Consisting of rituximab, alemtuzumab, ofatumumab, anti thymoglobulin, or gemtuzumab.

c Except for the leukocyte and neutrophil count, data on cell count were only available from 34 of the episodes with virus and 72 of the episodes without virus.

d No bacterial diagnostics were performed on patients without fever but are here assumed to be negative.

## Results

### Study population

A total of 159 neutropenic patients were included (Table 2); 123 patients with fever, and 36 unselected afebrile neutropenic patients. In case of participation at multiple neutropenic episodes only the first was considered in this study. Of the 123 febrile patients, 15 declined NPA and another one declined collection of extra blood samples. The afebrile patients were sampled completely. Thus, a total of 123 patients were included after presenting with febrile neutropenia and study samples for virus analysis were collected upon admission (27 patients) or within 24 hours (48 patients), within 48 hours (31 patients), and within 72 hours (17 patients) from admission.

### Viral findings

**Figure 1:**
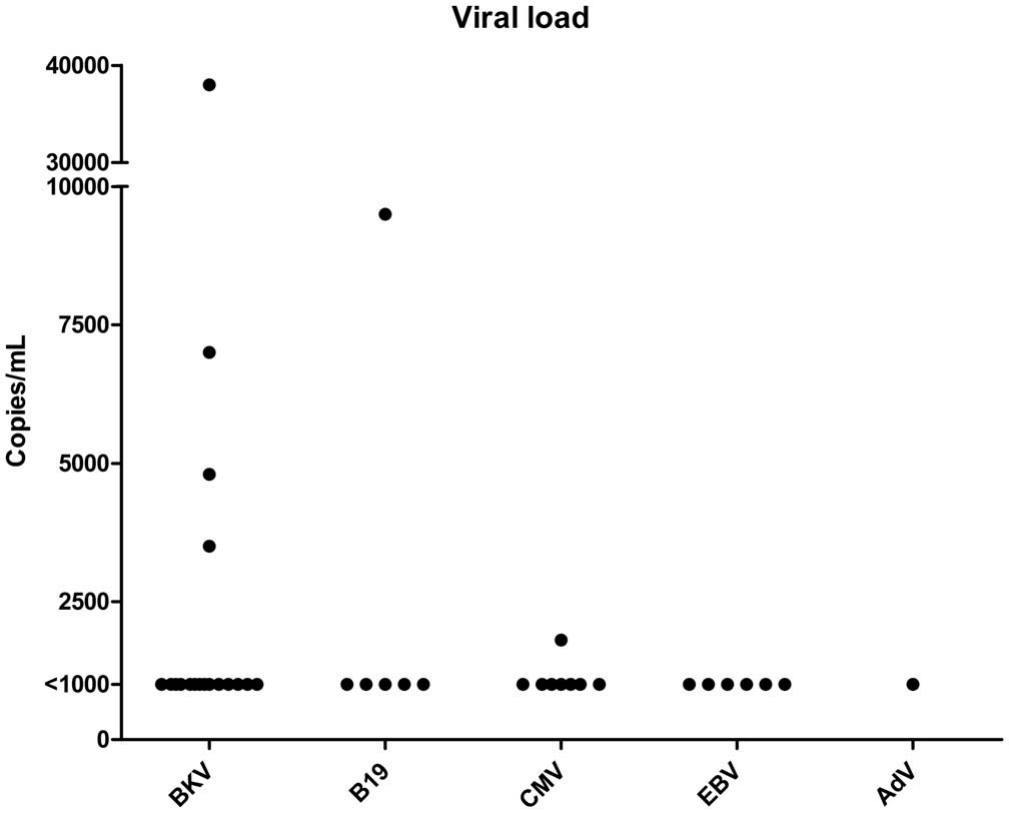
Titers of viruses detected in blood.

In a total of 56 patients (35%) at least one virus type was detected (Table 3). The detection rate was rather similar in blood (22%) and in NPA (18%). In blood, BKV dominated followed by CMV. In NPA HRV dominated followed by AdV. More than one virus type was simultaneously detected in nine (16%) of the 56 virus-positive patients and all except one were sampled upon fever. The viral load in blood samples was below 1 000 copies/mL with only the following exceptions; one CMV finding of 1800 copies/mL; four BKV findings of 3500, 4800, 7000 and 38000 copies/mL; and one B19 finding of 9500 copies/mL (Figure 1). All these were among the fever episodes.

**Table 3:** Presence of viruses in peripheral blood and nasopharynx in the 159 patients with neutropenia.

Specimen	Virus	No. of positive (%)		Co-existing viruses (n)^a^
Blood	BK polyoma virus (BKV)	18	(11.4)	B19 (1), EBV (1), IFB (1), HRV (2)
n = 158	Cytomegalovirus (CMV)	8	(5.1)	B19 (1), RSV (1), AdV in NPA+HRV (1)
	Parvovirus B19 (B19)	6	(3.8)	CMV (1), BK (1), EBV (1)
	Epstein-Barr virus (EBV)	6	(3.8)	BK (1), B19 (1)
	Adenovirus (AdV)	1	(0.6)	-
No. of patients with at least one virus detected in blood		35	(22.2)^b^	
				
NPA	Rhinovirus (HRV)	11	(7.6)	AdV in NPA+CMV (1), BK (2)
n = 144	Adenovirus (AdV)	6	(4.2)	HRV+CMV (1)
	Respiratory syncytial virus (RSV)	5	(3.5)	CMV (1)
	Influenza A virus (IFA)	1	(0.7)	-
	Influenza B virus (IFB)	2	(1.4)	BK (1)
	Metapneumovirus (HMPV)	2	(1.4)	-
No. of patients with at least one virus detected in NPA		26	(18.1)^c^	
No. of patients with at least one virus detected in any specimen		56	(35.2)	

**NOTE.** NPA, nasopharyngeal aspirate.

a All co-existing viruses are also listed in the column of viruses. Thus, they are presented, but not counted, twice.

b Percentage of total sampling occasions where blood was investigated.

c Percentage of total sampling occasions where NPA was investigated.

### Symptoms and signs of respiratory tract infection

Fifteen (58%) out of the 26 patients with a respiratory virus detected in NPA had URTS compared to 20 (17%) out of 118 patients without URTS (OR = 6.7, p<0.0001; Table 4). The predominating reported URTS was cough followed by rhinorrhea, sneezing or nasal congestion.

**Table 4:** Upper respiratory tract symptoms and fever in the 144 neutropenic patients where NPA were obtained.

	URTS	No URTS		Fever	No fever	OR (p)
Respiratory virus	(n = 35)	(n = 109)	OR (p)	(n = 109)	(n = 35)	
Rhinovirus	6	5^a^		10^a^	1^b^	
RSV	4	1		5	0	
Adenovirus	2	4^a^		6^a^	0	
Influenza A virus	0	1		1	0	
Influenza B virus	2	0		2	0	
Metapneumo virus	1	1		2	0	
Total (%)	15 (43)	11 (10)	6.7 (<.0001)	25 (23)	1 (3)	10 (<.01)

**NOTE.** URTS, upper respiratory tract symptoms; OR, odds ratio; RSV, respiratory syncytial virus.

a One case of co-infection of HRV and AdV.

b This patient did not have URTS either.

### Viral and bacterial findings in patients with febrile neutropenia

**Figure 2:**
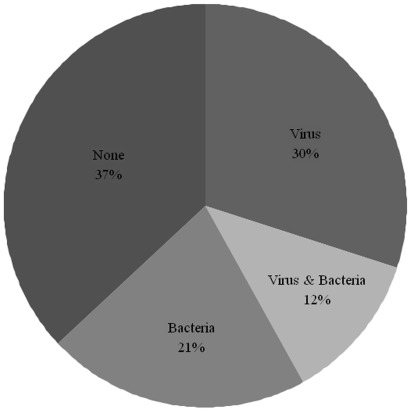
Documented microbiological pathogens in the 123 patients with febrile neutropenia.

As mentioned above, one or more viral pathogens were detected in 42% of the patients with febrile neutropenia. In 15 (29%) of these patients there was concurrently a documented bacterial infection. Conversely, bacterial infection was documented in 41 (33%) of the cases with febrile neutropenia and in 15 (37%) of these virus co-occurred (Figure 2). There were no statistical significant differences between the groups regarding C-reactive protein (CRP) levels at fever onset or number of days with fever. However, the number of days with intravenous antibiotics was higher for patients with documented bacterial infection (median 9 days, range 4–24) compared to those with virus only (median 6, range 2–17) or no pathogen found (median 6, range 3–15; p<0.01 and p<0.001, respectively).

### Characteristics and clinical parameters of patients with and without virus

Chronic lymphocytic leukemia (CLL) was overrepresented in the virus-positive group (11% vs. 1%, p<0.01; Table 2) and, even though border line significant, viruses were more likely to be detected among non-Hodgkin lymphoma (NHL) patients (45% vs. 28%, p = 0.05). In contrast, few recipients of autologous HSCT were virus positive (3.6% vs. 26%, p<0.001). Regarding the latest treatment for the underlying disease, both steroid treatment and monoclonal antibody therapy were associated with virus findings, and eight of eleven given the combined drugs were positive. The absolute counts of cell populations examined were lower in the virus-infected group (Table 2). However, the differences did not reach statistical significance with the exception of the CD4^+^ T cell count. The patients on prophylactic treatment with aciclovir were underrepresented in the virus-positive group (38% vs. 56%, p<0.05). A total of 52 (42%) out of the 123 patients with febrile neutropenia had virus detected, compared to 4 (11%) out of the 36 patients without fever (OR = 5.9, p<0.001). This was true for viruses detected in blood (OR = 3.9, p<0.05) as well as viruses detected in NPA (OR = 11, p<0.01; Table 4). As reactivation of viruses in blood is associated with immunosuppression, we performed univariate analyses of the characteristics in table 2 regarding the febrile and non-febrile patients. There were no differences regarding gender, age, or underlying disease. However, 117 (95%) of the febrile patients and only 30 (83%) of the afebrile patients had antineoplastic drugs included in their latest treatment (p<0.5), and there was also a difference in the use of steroids (30% vs. 11%, p<0.05). The cell count for all populations except monocytes were significantly higher in the afebrile patients (p<0.001). After multivariate regression analysis with presence of virus dependent variable and parameters in Table 2 with a p value less than 0.10 as covariates, CLL and fever remained as independently associated to presence of virus (OR = 7.5, p<0.01 and OR = 13, p<0.0001), respectively. There were no reports on hemorrhagic cystitis in the cases of detectable BKV. Furthermore, we were unable to show an association of elevated serum levels of creatinine and presence of BKV (data not shown).

## Discussion

Using highly sensitive methods to identify a broad range of viruses, we have estimated the prevalence of respiratory viruses and viremia in hematological patients with neutropenia other than allogeneic HSCT recipients. Viral infections were commonly detected in patients with CLL and NHL. Among subpopulations of immunological cell types investigated, decline in the CD4^+^ T cell count was associated with these infections. Viral infections were associated with fever, and specifically respiratory viruses in NPA were associated with URTS. Although the majority of viral infections did not co-occur with bacterial infection, viral and bacterial co-infections were common.

The proportion of different virus species in NPA differed from an earlier large study by Chemaly et al on adult hematological patients [23]. HRV was the commonest finding in our study which could be explained by the use of PCR instead of culture. Moreover, the study by Chemaly et al was retrospective and only included patients with documented positive viral culture. However, our findings in NPA were comparable to recent studies on children with febrile neutropenia [5], [24] where PCR was used. Moreover, with the exception of corona virus and PIV, the proportions of viral species corresponded well to a study on community acquired pneumonia in the same geographical area [25]. The latter confirms an earlier finding that the temporal occurrence of viral infections in immunocompromised patients tends to mirror their occurrence in the community [15].

Approximately half of the viral findings were viremia with BKV and CMV as dominating pathogens. A comparable screening in young children with febrile neutropenia showed lower frequency [5], but this discrepancy is probably explained by the fact that the prevalence of latent viral infections increases with age. It has been shown that allogeneic transplanted patients with a BK viral load of more than 10^4^ copies/mL had a significantly higher risk of having hemorrhagic cystitis than patients with a viral load of less than 10^4^ copies/mL [26]. In a recent pediatric study on a post-HSCT population, the degree of BK viremia seemed to predict renal, urologic, and overall outcome [27]. However, in our less immunosuppressed cohort with mostly very low titers, we had no reports on symptoms of disease associated to BKV.

Although we found an independent association between viral findings and fever, one should interpret the results with caution because of the discrepancy in grade of immunosuppression between the febrile and afebrile patients. Furthermore, multivariate analysis with this low number of patients carries the risk of small sample bias. Even though the levels of virus in blood were lower than those expected from symptomatic disease, the independent association prompts the question whether the viral load was sufficient to generate fever. Furthermore, not all viral respiratory tract infections cause fever, and different virus types are known to be variously pyrogenic [28]. In our study, not all patients with fever were completely sampled for detection of viruses. We thus risk underestimating the association between fever and viral findings. Although bacterial culture is limited in sensitivity, it is of interest to note that virus existed more often as the sole pathogen identified here than as a cofactor with bacteria. The fact that PCR is a highly sensitive method always raises the question of the findings' relevance. We did, however, determine an association between respiratory viruses and URTS, indicating symptomatic infection.

Until recently, viral infections were regarded as a concern in hematological malignancies primarily in patients who had undergone allogeneic HSCT. However, in the last years, the use of monoclonal antibodies directed against B and/or T cells for treatment of hematological malignancies such as NHL and CLL has had potential to increase the incidence of viral infections in these groups as well. These two diagnoses as well as receiving monoclonal antibody therapy were associated to detection of virus in our material. After multivariate analysis, only CLL showed to be independently associated, but once again, with risk of small sample bias.

In allogeneic HSCT recipients, lymphocytopenia is a known risk factor for the development of lower respiratory tract infection [29], and reconstitution of the lymphocyte population is known to reduce the risk for viremia with CMV and AdV [30], [31]. In our study, all measured cell counts were lower in the virus-positive patients but only the difference in CD4^+^ cell count was statistically significant. The function of lymphocytes can be altered by both the treatment and the underlying disease [32] and it would therefore be interesting to investigate an association between not only the cell count but also the cell function to risk of viral infection in this patient category.

In conclusion, both respiratory virus infections and low titers of viruses in blood from patients with neutropenia were common. Patients with CLL and patients with fever were independently associated to these infections, and viral findings in NPA were associated to URTS indicating active infection. Although this is a small study in a heterogeneous group of hematological patients, it is possible that a proportion of episodes of febrile neutropenia could originate from virus infections. Larger prospective longitudinal studies should be conducted to investigate the possible causality between neutropenic fever and viral infections.
